# P-2078. Performance of Anyplex and Xpert for the Detection of *Mycobacterium tuberculosis* complex in Respiratory and Non-Respiratory Samples

**DOI:** 10.1093/ofid/ofae631.2234

**Published:** 2025-01-29

**Authors:** Alina Gaxiola-Castro, Samantha Flores-Treviño, Paola Bocanegra Ibarías, Adrian Camacho-Ortiz

**Affiliations:** Department of Infectious Diseases, Hospital Universitario Dr. José Eleuterio González Facultad de Medicina, Universidad Autónoma de Nuevo León, Monterrey, México, Monterrey, Nuevo Leon, Mexico; Universidad Autónoma de Nuevo León, Monterrey, Nuevo Leon, Mexico; Universidad Autónoma de Nuevo León, Monterrey, Nuevo Leon, Mexico; Department of Infectious Diseases, Hospital Universitario Dr. José Eleuterio González Facultad de Medicina, Universidad Autónoma de Nuevo León, Monterrey, México, Monterrey, Nuevo Leon, Mexico

## Abstract

**Background:**

Tuberculosis diagnosis requires rapid and sensitive diagnostic methods. The aim was to evaluate the performance of Anyplex MTB/NTM and Xpert MTB/RIF assays for the detection of *Mycobacterium tuberculosis* complex (MTC) in respiratory and non-respiratory samples from patients suspected of having tuberculosis in a third-level hospital in Mexico.

**Figure:**
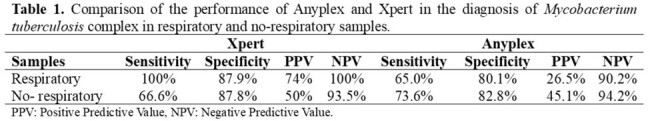

**Methods:**

Samples (n=436) from January 2020 to December 2023 in a hospital from Monterrey, Mexico underwent paired microbiological (MGIT or Lowenstein-Jensen culture) and molecular assays (Anyplex™ MTB/NTM Real-time Detection or Xpert MTB/RIF™) for mycobacterial detection.

**Results:**

Of the 279 (63.8%) respiratory samples (bronchoalveolar lavage 71.3%, sputum 20.7% and tracheal aspirate 7.8%), positive culture and detected PCR were found in 26/201 samples by Anyplex and in 20/78 samples by Xpert whereas negative culture and detected PCR were found in 32 and 7 samples, respectively. Anyplex showed 65% sensitivity, 80.1% specificity, 26.5% positive predictive value (PPV), and 90.2% negative predictive value (NPV) whereas Xpert showed 100% sensitivity, 87.9% specificity, 74% PPV and 100% NPV. Of the 157 (36.1%) non-respiratory samples (cerebrospinal fluid 37.3%, soft tissues 21.5%, pleural 22.1%, lymph nodes 11.3%, and synovial 7.5%), positive culture and detected PCR were found in 14/118 samples by Anyplex and in 4/39 by Xpert whereas negative culture and detected PCR were found in 17 and 4 samples, respectively. Anyplex showed 73.6% sensitivity, 82.8% specificity, 45.1% PPV, 94.2% NPV whereas Xpert showed 66.6% sensitivity, 87.8% specificity, 50% PPV and 93.5% NPV.

**Conclusion:**

Xpert assays showed higher sensitivity than Anyplex for the detection of MTC in respiratory samples and showed higher specificity compared to Anyplex in both respiratory and non- respiratory samples. Xpert showed higher PPV for respiratory samples, indicating its superiority disregarding true positive cases.

**Disclosures:**

All Authors: No reported disclosures

